# Cardiovascular Health, as per Life's Essential 8, and Impaired Lower-Extremity Function in Older Adults

**DOI:** 10.14336/AD.2025.0347

**Published:** 2026-06-13

**Authors:** David Gómez-Ángel, Mercedes Sotos-Prieto, David Martínez-Gómez, Auxiliadora Graciani, Esther García-Esquinas, Fernando Rodríguez-Artalejo, Rosario Ortolá

**Affiliations:** ^1^Department of Preventive Medicine and Public Health, Universidad Autónoma de Madrid, Madrid, Spain.; ^2^CIBER of Epidemiology and Public Health (CIBERESP), Madrid, Spain.; ^3^Department of Environmental Health, Harvard T.H. Chan School of Public Health. Boston, MA, USA.; ^4^IMDEA Food Institute. CEI UAM+CSIC, Madrid, Spain.; ^5^Department of Chronic Diseases, National Center for Epidemiology, Carlos III Health Institute, Madrid, Spain

**Keywords:** Life's Essential 8, Short Physical Performance Battery, Cohort study, Aging

## Abstract

Cardiovascular health (CVH) is a broad construct that encompasses multiple behavioral and biological factors. A decline in CVH has been associated with various adverse health outcomes, but its role in impaired lower-extremity function (ILEF), a major contributor to disability, diminished quality of life and mortality in older adults, is unknown. Therefore, we examined the cross-sectional and prospective association between CVH and lower-extremity function. Using data from 2,487 individuals aged ≥65y from the Seniors-ENRICA-2 cohort, we estimated CVH at baseline using the American Heart Association’s Life's Essential 8 (LE8) score (range 0 to 100, with higher values indicating better CVH). We assessed ILEF at baseline and at 2.4-year and 5.2-year follow-ups using the Short Physical Performance Battery (SPPB). Statistical analyses were conducted with logistic regression with adjustment for the main confounders. ILEF was present in 26.8% of participants at baseline (666 events). The cumulative incidence over 2.4 and 5.2 years was 24.8% (278 events) and 22.5% (157 events), respectively. A 10-point higher LE8 score at baseline was associated with lower prevalence of ILEF (SPPB ≤9) at baseline (odds ratio [OR] 0.75, 95% confidence interval [CI]: 0.69-0.80), and lower risk of incident ILEF over 2.4 years (OR 0.77, 95% CI: 0.68-0.87) and 5.2 years (OR 0.76, 95% CI: 0.65-0.89). Physical activity, glucose levels, body mass index and nicotine exposure stood out as major contributors to the lower risk of incident ILEF associated with a higher LE8 score. A higher LE8 score was associated with both a lower prevalence and incidence of ILEF in older adults. Comprehensive evaluation of CVH offers insight into older adults' lower-extremity function and how it may progress over time, identifying opportunities for early intervention.

## INTRODUCTION

In most of the world, people are living longer lives, resulting in a rapid increase in both the number and percentage of the population aged 65 years and over. According to the United Nations Department of Economic and Social Affairs (2020), this segment of the population is projected to double in the next three decades, reaching over 1.5 billion people worldwide by 2050 [1]. Two thirds of people over 65 years old are affected by multimorbidity, which is strongly associated with impaired quality of life, disability, dependence, and death [2]. Cardiovascular disease (CVD) is the leading contributor to burden of disease in the older adult, partly due to its high risk of causing physical limitations [2]. Since most cases of CVD are not curable, prevention is paramount, either by avoiding the onset of major risk factors or by controlling those already present.

Cardiovascular health (CVH) is a broad construct that includes both socio-behavioral and biological components, as outlined in the Life's Essential 8 (LE8) model by the American Heart Association (AHA) in 2022 [3], which updated the Life's Simple 7 (LS7) [4] developed in 2010 to assess and promote CVH. This newer model revised the measurement methods used in the LS7 for the health behaviors domain and added sleep health as a new CVH metric. LE8 comprises 4 behavioral factors, namely diet quality, nicotine exposure, physical activity and sleep health, and 4 objective health factors, namely body mass index (BMI), blood lipids, blood glucose and blood pressure. In addition, some researchers have proposed incorporating psychological health into the AHA's CVH metric, updating it from the LE8 to the Life's Crucial 9 [5]. This highlights the ever-evolving nature of the CVH concept.

Still, since the adoption of CVH metrics, several associations have been found between poor CVH and adverse health outcomes. These include subclinical atherosclerosis [6], CVD, and all-cause mortality [7, 8]. Additionally, poor CVH contributes to the development of frailty [9], premature cardiovascular events [10], heart failure [11], lower perception of general health, decreased productivity at work, and lower health-related quality of life [12]. However, the role of CVH in impaired lower-extremity function (ILEF), a major contributor to falls, sarcopenia, frailty, postoperative complications, CVD, institutionalization and mortality in older adults [13], is unknown. Therefore, we aimed to investigate the cross-sectional and prospective association between CVH, as estimated by the AHA's LE8 score, and lower-extremity function in older adults.

## MATERIALS AND METHODS

### Study population

We used data from the Seniors-ENRICA-2 cohort, composed of 3,273 community-dwelling individuals aged ≥ 65 years, enrolled between 2015 and 2017 (baseline) in the metropolitan area of Madrid, Spain. Data regarding socio-demographics, health status, and lifestyle were gathered via a telephone survey. Following this, two in-home visits were carried out to gather biological specimens, conduct physical examinations, and evaluate dietary habits using a diet history [14]. Subsequently, participants were invited to update the study information in 2018-19 (2.4-year follow-up, range: 1.6-3.3) and 2022-23 (5.2-year follow-up, range: 4.4-6.7) [15].

The study protocol was approved by the Clinical Research Ethics Committee of *La Paz* University Hospital, in Madrid, Spain, and all study participants provided written informed consent.

### Study variables

#### Cardiovascular health assessment using the AHA’s Life’s Essential 8

LE8 scoring was calculated following the AHA instructions [3], where each of the 4 health behaviors (diet, physical activity, nicotine exposure, sleep health) and 4 health factors (BMI, blood lipids, blood glucose, blood pressure) were graded on a scale from 0 to 100 points (best CVH). At the baseline visit, habitual food consumption in the preceding year was estimated with a validated diet history developed from the one used in the EPIC cohort study in Spain [16], and the diet metric was assessed using the Alternative Healthy Eating Index (AHEI) [17]. Physical activity was estimated with the validated EPIC Physical Activity questionnaire [18] and the physical activity metric was calculated as the sum of minutes spent in do-it-yourself activities, gardening, sports, cycling, and going up stairs [19]. The last three activities considered vigorous, so their time was counted double. Nicotine exposure was calculated using self-reported cigarette use. Sleep health was also assessed using self-reported average nighttime sleep [20]. BMI was calculated as the ratio of measured body weight to height squared, obtained in the physical examination at the home visit. Blood lipid score was assessed using serum total and HDL cholesterol measured in samples collected at the first home visit, with non-HDL cholesterol calculated. Blood glucose was measured using fasting blood glucose and HbA1c at the first home visit. The laboratory measurements were performed centrally at the Department of Laboratory Medicine of the *La Paz* University Hospital in Madrid. Blood pressure (BP) was measured using standardized procedures with validated automatic equipment (model Omron M6)[21]. In the analyses, BP was calculated as the mean of at least 3 of the last 5 readings, excluding the first reading to minimize the alarm response during BP determination. The LE8 overall score was calculated by averaging the individual component scores, resulting in a composite score on a 0-100 scale, consistent with the scale used for each individual metric. The definitions and classifications for each metric in our study can be found in Supplementary Table 1 in the supplement.

#### Lower-extremity function assessment with the Short Physical Performance Battery (SPPB)

During the baseline and the follow up visits, we recorded performance on the three tests that compose the SPPB: balance, lower-extremity power, and gait speed. Balance was tested by the ability to stand upright for 10 seconds in three different positions: side-by-side stand, semi-tandem stand, and tandem stand. Power was assessed by the ability of the individual to stand up from a chair 5 consecutive times. Time was recorded and used to calculate the final score. Gait speed was tested by measuring the time required to walk 2.44 meters at a normal pace. Cutoff points for each test were set according to the SPPB original article [22] and scored from 0 (worst) to 4 points (best). Overall scores were calculated as the sum of the 3 tests and range from 0 points (worst) to 12 points (best).

We then categorized study participants as those with either high (i.e., >9) or low (i.e., ≤9) SPPB score, which in turn represents either preserved or impaired lower-extremity function, respectively. This cut-off value has been shown to predict frailty, sarcopenia and mobility disability in older adults [23-26].

#### Other variables

At baseline, we also obtained information on potential confounders of the study associations. We included age, sex, and educational level (categorized as primary or less, secondary and university) as sociodemographic factors. For lifestyle habits, we included time watching TV, energy intake and alcohol consumption (categorized as never, former, moderate, or heavy drinkers). Additionally, the self-reported physician-based diagnoses of CVD, respiratory disease, musculoskeletal disease, cancer, and depression requiring treatment were included as clinical characteristics. Information regarding the medications used, through self-reports, which were verified against the medication packages at home, was also collected.

**Table 1:** Baseline characteristics of study participants by LE8 category.

	Overall	Low LE8 (<50)	Moderate LE8 (50-79)	High LE8 (≥80)	p-value^a^
**n (%)**	2,487 (100)	387 (15.56)	1,877 (75.47)	223 (8.97)	
**Age (years)**	71.54 (4.38)	71.71 (4.79)	71.51 (4.34)	71.46 (4.02)	0.968
**Sex, n (%)**					0.433
**Male**	1,173 (47.17)	185 (47.80)	892 (47.52)	96 (43.05)	
**Female**	1,314 (52.83)	202 (52.20)	985 (52.48)	127 (56.95)	
**Educational level, n (%)**					<0.001
**Primary or less**	1,581 (63.57)	280 (72.35)	1,173 (62.46)	128 (57.40)	
**Secondary**	464 (18.66)	60 (15.50)	363 (19.34)	41 (18.39)	
**University**	442 (17.77)	47 (12.14)	341 (18.17)	54 (24.22)	
**Time watching TV (h/day)**	3.17 (1.56)	3.67 (1.81)	3.11 (1.52)	2.80 (1.25)	<0.001
**Energy intake (kcal/day)**	1949 (354)	2016 (435)	1942 (338)	1893 (318)	0.003
**Alcohol consumption, n (%)**					0.008
**Never drinker**	468 (18.82)	84 (21.71)	339 (18.06)	45 (20.18)	
**Moderate drinker**	1,318 (53.00)	174 (44.96)	1,024 (54.56)	120 (53.81)	
**Heavy drinker**	544 (21.87)	101 (26.10)	405 (21.58)	38 (17.04)	
**Former drinker**	157 (6.31)	28 (7.24)	109 (5.81)	20 (8.97)	
**Cardiovascular disease diagnosis, n (%)**	83 (3.34)	18 (4.65)	57 (3.04)	8 (3.59)	0.267
**Respiratory disease diagnosis, n (%)**	194 (7.80)	43 (11.11)	140 (7.46)	11 (4.93)	0.013
**Musculoskeletal disease diagnosis, n (%)**	1,120 (45.03)	177 (45.74)	835 (44.49)	108 (48.43)	0.511
**Cancer diagnosis, n (%)**	71 (2.85)	10 (2.58)	57 (3.04)	4 (1.79)	0.625
**Depression diagnosis, n (%)**	204 (8.20)	45 (11.63)	146 (7.78)	13 (5.83)	0.017
**ILEF (SPPB ≤ 9), n (%)**	666 (26.78)	162 (41.86)	468 (24.93)	36 (16.14)	<0.001

Note. ILEF, impaired lower extremity function; LE8, Life's Essential 8; SPPB, Short Physical Performance Battery. Values are means (standard deviations) unless otherwise specified.

a For categorical variables we used Chi squared tests; for continuous variables we used ANOVA, Welch's t-tests or Kruskal-Wallis tests, as appropriate.

### Statistical analysis

The initial sample consisted of 3,273 participants, from which we excluded 775 due to missing data on at least one LE8 component, 6 due to a lack of at least one of baseline SPPB tests, and 5 for missing values on at least one confounder. Therefore, 2,487 participants were included in the cross-sectional analysis. For the prospective analysis, 1,482 participants had updated SPPB scores at the 2.4-year follow-up and 881 had the updated scores at the 5.2-year follow-up (Supplementary Fig. 1). Compared to participants included in the analyses, those excluded from the cross-sectional analysis and those lost to follow-up were older, spent more time watching TV, and had a higher prevalence of CVD and ILEF. Additionally, participants lost to follow-up were less educated, less likely to be current drinkers, and had a higher prevalence of musculoskeletal disease (Supplementary Table 2

**Table 2:** Odds ratios (95% confidence interval) for the cross-sectional, 2.4-year prospective and 5.2-year prospective association of LE8 score with impaired lower-extremity function (SPPB ≤9).

	Low LE8 (<50)	Moderate LE8 (50-79)	High LE8 (≥80)	p-trend	Per 10 points
**Cross-sectional**					
No. cases/N	162/387	468/1877	36/223		666/2487
Model 1	Ref.	0.46 (0.36; 0.58)***	0.26 (0.17; 0.40)***	<0.001	0.73 (0.68; 0.79)***
Model 2	Ref.	0.48 (0.37; 0.61)***	0.28 (0.18; 0.43)***	<0.001	0.74 (0.69; 0.80)***
Model 3	Ref.	0.48 (0.38; 0.62)***	0.28 (0.18; 0.43)***	<0.001	0.75 (0.69; 0.80)***
**2.4-year prospective**					
No. cases/N	39/125	220/865	19/131		278/1121
Model 1	Ref.	0.70 (0.46; 1.08)	0.31 (0.16; 0.59)***	<0.001	0.77 (0.68; 0.86)***
Model 2	Ref.	0.74 (0.48; 1.16)	0.32 (0.17; 0.62)***	<0.001	0.77 (0.69; 0.87)***
Model 3	Ref.	0.74 (0.47; 1.16)	0.31 (0.16; 0.61)***	<0.001	0.77 (0.68; 0.87)***
**5.2-year prospective**					
No. cases/N	27/74	115/530	15/95		157/699
Model 1	Ref.	0.43 (0.25; 0.74)**	0.26 (0.12; 0.55)***	<0.001	0.75 (0.65; 0.87)***
Model 2	Ref.	0.43 (0.24; 0.75)**	0.25 (0.12; 0.56)***	<0.001	0.75 (0.65; 0.88)***
Model 3	Ref.	0.43 (0.24; 0.77)**	0.25 (0.11; 0.56)***	<0.001	0.76 (0.65; 0.89)***

Note. LE8: Life's Essential 8. SPPB, Short Physical Performance Battery.

Model 1: Logistic regression model adjusted for: age, sex, and education.

Model 2: Further adjusted for time watching TV, energy intake and alcohol consumption.

Model 3: Further adjusted for cardiovascular disease, respiratory disease, musculoskeletal disease, cancer and depression.

*p<0.05; **p<0.01; ***p<0.001

The cross-sectional and prospective associations between CVH and ILEF were evaluated using logistic regression, with CVH modeled as: 1) continuous LE8 score (per 10-point increment on a 100-point scale), 2) LE8 score categories (low, i.e., <50; moderate, i.e., 50-79; high, i.e., ≥80), and 3) restricted cubic splines with knots at the 10th, 50th and 90th percentiles to evaluate potential nonlinear relationships. Potentially important confounders were included in three hierarchical models: the first one adjusted for sociodemographic factors, the second further adjusted for lifestyle habits, and the third additionally adjusted for clinical characteristics. Results were summarized with odds ratios and their 95% confidence intervals. The same approach was used to assess the associations between CVH and each component of the SPPB score, considering impaired function as a score ≤3, and to examine the associations between each LE8 metric and ILEF.

To examine if chronic disease onset modified the association between baseline LE8 and incident ILEF, we conducted a stratified analysis based on incident chronic disease over follow-up and tested an interaction term defined as the product of LE8 × incident chronic disease. Also, we ran an additional model further adjusting for the change in LE8 score between baseline and follow-up to account for potential behavior changes following disease diagnosis that may confound the primary association.

Finally, we examined if sociodemographic, lifestyle, and clinical variables modified the study associations by testing interaction terms defined as the product between the overall LE8 score and such variables.

Statistical significance was set at a 2-sided p-value <0.05. Analyses were performed using Stata®, version 18 (StataCorp).

## RESULTS

The LE8 score was low in 15.6% of study participants, moderate in 75.5% and high in 9.0%. Individuals with worse CVH were more likely to be less educated, to spend more time watching TV, to consume more calories, to have diagnoses of respiratory disease, and depression, and to present ILEF. They also were less frequently moderate drinkers and more frequently heavy drinkers (Table 1).

**Figure 1.**
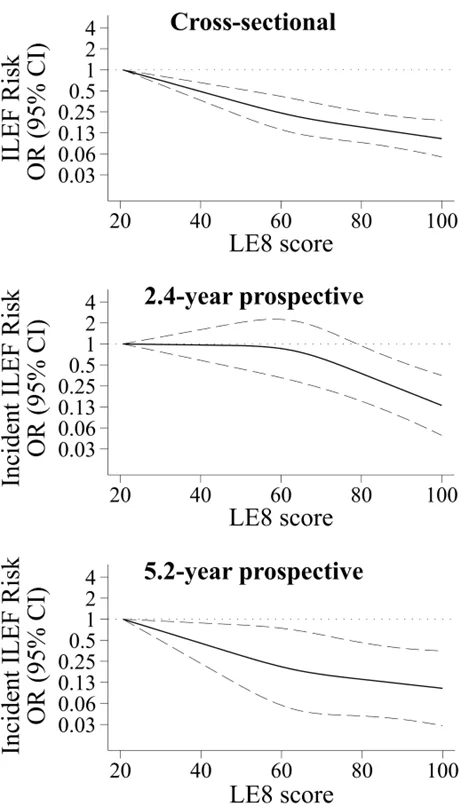
**Dose-response association of global Life's Essential 8 score (LE8) with the risk of impaired lower-extremity function (SPPB ≤ 9)**. Note. Restricted cubic spline logistic regression model adjusted for age, sex, education, time watching TV, energy intake, alcohol consumption, cardiovascular disease, respiratory disease, musculoskeletal disease, cancer and depression

At baseline, impaired lower-extremity function (ILEF; SPPB ≤9) was present in 26.8% of participants (666 events). The cumulative incidence was 24.8% (278 events) and 22.5% (157 events) at 2.4 and 5.2 years of follow-up, respectively. A higher overall LE8 score at baseline was associated with lower prevalence of ILEF at baseline and lower risk of incident ILEF in the 2.4-year and 5.2-year prospective analyses (Fig. 1). In the fully adjusted model, each 10-point increase in the LE8 score was associated with a 25% lower prevalence of ILEF, and a 23% and 24% lower risk of ILEF during the 2.4-year and 5.2-year follow-up, respectively (Table 2). Corresponding decrease in ILEF for high vs. low LE8 score were 72%, 69%, and 75%. Likewise, having a moderate vs. low LE8 score was associated with 52% lower prevalence of ILEF at baseline and a 57% lower incidence at the 5.2-year follow-up, whereas a non-significant tendency towards lower risk of ILEF was noted in the 2.4-year follow-up (Table 2).

When analyzing individual components, each 10-point increase in the overall LE8 score was associated with a 33%, 11%, and 20% lower baseline prevalence of low balance, low gait speed and low power, respectively. Corresponding risk reductions in the 2.4-year follow-up were 18% for low balance and 21% for low gait speed, while in the 5.2-year follow-up, they were 34% for low balance and 15% for low gait speed (Table 3). The dose-response associations are presented in Supplementary Supplementary Figure 2.

Regarding LE8 health behavior components, a 10-point higher physical activity score was linked to a 7%, 6%, and 5% lower frequency of ILEF in the cross-sectional, 2.4-year and 5.2-year follow-up, respectively. Diet and sleep were also associated with ILEF at baseline, but this association disappeared during the follow-up, while lower nicotine exposure was associated with an 8% lower risk of ILEF at the 5.2-year follow-up only (Table 4).

Among LE8 health factors components, for each 10-point increase in the BMI score, there was 11% lower frequency of ILEF, both at the cross-sectional and 5.2-year follow-up timepoints; and for the blood glucose score, ILEF was 7% less frequent both at the cross-sectional and 2.4-year follow-up timeframes. No association was found between blood lipids or blood pressure and ILEF (Table 4).

The association between LE8 and ILEF did not significantly differ by chronic disease incidence status (Supplementary Table 3) and was similar to the observed in the main analysis when further adjusting for changes in the LE8 score over the follow-up (Supplementary Table 4). Sociodemographic, lifestyle, and clinical variables did not modify the study associations (Supplementary Figures 3, 4, and 5).

## DISCUSSION

In this cohort of Spanish older adults, having a better CVH, as measured by the AHA’s LE8, was inversely associated with ILEF both cross-sectionally and over 2.4-year and 5.2-year follow-ups. This association was linear, with incremental protection with higher LE8 scores, and robust to adjustment for sociodemographic, lifestyle and clinical characteristics. Physical activity, glucose levels, BMI and nicotine exposure stood out as major contributors to the lower risk of incident ILEF associated with better CVH.

Because the LE8 score represents the average score across eight cardiovascular health components, a 10-point increase can result either from moderate improvements across multiple behaviors or from a major improvement in a single domain. For example, increasing physical activity from 30-59 to 60-89 minutes per week, extending sleep duration from 5-6 to 7-8 hours per night, and improving diet quality from the 25th-49th percentile to the 50th-74th percentile could collectively raise the LE8 score by approximately 10 points. Alternatively, a major improvement in a single factor, such as strict control of previously poorly managed diabetes, hypertension, or hyperlipidemia, could individually result in a 10-point gain in the overall LE8 score.

**Table 3:** Odds ratios (95% confidence interval) for the cross-sectional, 2.4-year prospective and 5.2-year prospective association of LE8 score with individual SPPB components.

	Low LE8 (<50)	Moderate LE8 (50-79)	High LE8 (≥80)	p-trend	Per 10 points
**CROSS-SECTIONAL**					
**Balance test score ≤3**					
No. cases/N	94/387	211/1877	14/223		319/2487
Model 1	Ref.	0.38 (0.29; 0.51)***	0.20 (0.11; 0.37)***	<0.001	0.66 (0.60; 0.73)***
Model 2	Ref.	0.39 (0.29; 0.53)***	0.21 (0.11; 0.39)***	<0.001	0.67 (0.60; 0.74)***
Model 3	Ref.	0.40 (0.29; 0.54)***	0.20 (0.11; 0.38)***	<0.001	0.67 (0.61; 0.74)***
**Gait speed test score ≤3**					
No. cases/N	137/387	562/1877	55/223		754/2487
Model 1	Ref.	0.81 (0.64; 1.02)	0.62 (0.42; 0.90)*	0.010	0.89 (0.83; 0.95)***
Model 2	Ref.	0.80 (0.63; 1.02)	0.62 (0.42; 0.90)*	0.010	0.88 (0.82; 0.94)***
Model 3	Ref.	0.81 (0.64; 1.04)	0.63 (0.43; 0.92)*	0.015	0.89 (0.83; 0.95)***
**Chair-stand test score ≤3**					
No. cases/N	346/387	1413/1877	160/223		1919/2487
Model 1	Ref.	0.37 (0.26; 0.52)***	0.32 (0.20; 0.50)***	<0.001	0.78 (0.72; 0.85)***
Model 2	Ref.	0.39 (0.27; 0.55)***	0.33 (0.21; 0.52)***	<0.001	0.79 (0.73; 0.86)***
Model 3	Ref.	0.39 (0.28; 0.55)***	0.34 (0.21; 0.53)***	<0.001	0.80 (0.73; 0.86)***
**2.4-YEAR PROSPECTIVE**					
**Balance test score ≤3**					
No. cases/N	15/161	100/1021	7/144		122/1326
Model 1	Ref.	0.99 (0.55; 1.78)	0.43 (0.17; 1.11)	0.111	0.82 (0.70; 0.95)**
Model 2	Ref.	1.06 (0.58; 1.91)	0.46 (0.18; 1.19)	0.156	0.83 (0.71; 0.97)*
Model 3	Ref.	1.02 (0.56; 1.85)	0.43 (0.16; 1.13)	0.122	0.82 (0.71; 0.96)*
**Gait speed test score ≤3**					
No. cases/N	67/139	288/792	34/116		389/1047
Model 1	Ref.	0.61 (0.41; 0.89)*	0.41 (0.24; 0.72)**	0.001	0.79 (0.71; 0.87)***
Model 2	Ref.	0.62 (0.42; 0.92)*	0.43 (0.25; 0.75)**	0.003	0.79 (0.71; 0.88)***
Model 3	Ref.	0.62 (0.42; 0.93)*	0.42 (0.24; 0.73)**	0.002	0.79 (0.71; 0.88)***
**Chair-stand test score ≤3**					
No. cases/N	14/28	143/311	14/44		171/383
Model 1	Ref.	0.74 (0.34; 1.64)	0.37 (0.14; 1.02)	0.039	0.88 (0.74; 1.06)
Model 2	Ref.	0.74 (0.33; 1.67)	0.32 (0.11; 0.92)*	0.023	0.86 (0.72; 1.04)
Model 3	Ref.	0.76 (0.33; 1.75)	0.35 (0.12; 1.02)	0.040	0.89 (0.74; 1.08)
**5.2-YEAR PROSPECTIVE**					
**Balance test score ≤3**					
No. cases/N	8/87	31/613	3/106		42/806
Model 1	Ref.	0.36 (0.15; 0.87)*	0.16 (0.04; 0.67)*	0.007	0.62 (0.47; 0.80)***
Model 2	Ref.	0.39 (0.15; 0.98)*	0.20 (0.04; 0.86)*	0.022	0.65 (0.49; 0.86)**
Model 3	Ref.	0.39 (0.15; 1.01)	0.19 (0.04; 0.86)*	0.023	0.66 (0.49; 0.88)**
**Gait speed test score ≤3**					
No. cases/N	55/81	305/507	49/89		409/677
Model 1	Ref.	0.73 (0.44; 1.21)	0.58 (0.30; 1.09)	0.093	0.84 (0.74; 0.95)**
Model 2	Ref.	0.75 (0.44; 1.25)	0.59 (0.31; 1.14)	0.121	0.85 (0.75; 0.97)*
Model 3	Ref.	0.74 (0.44; 1.25)	0.57 (0.29; 1.10)	0.093	0.85 (0.75; 0.97)*
**Chair-stand test score ≤3**					
No. cases/N	7/18	67/210	12/37		86/265
Model 1	Ref.	0.57 (0.20; 1.60)	0.49 (0.14; 1.69)	0.324	0.93 (0.74; 1.18)
Model 2	Ref.	0.45 (0.15; 1.30)	0.34 (0.09; 1.25)	0.146	0.87 (0.68; 1.11)
Model 3	Ref.	0.51 (0.17; 1.57)	0.40 (0.10; 1.53)	0.224	0.89 (0.69; 1.15)

Note. LE8: Life's essential 8; SPPB: Short Physical Performance Battery

Model 1: Logistic regression model adjusted for: age, sex, and education.

Model 2: Further adjusted for time watching TV, energy intake and alcohol consumption.

Model 3: Further adjusted for cardiovascular disease, respiratory disease, musculoskeletal disease, cancer and depression.

*p<0.05; **p<0.01; ***p<0.001

**Table 4:** Odds ratios (95% confidence interval) for the cross-sectional, 2.4-year prospective and 5.2-year prospective association between each LE8 component score (per 10 points) and impaired lower-extremity function (SPPB ≤9).

	Cross-sectional	2.4-year follow-up	5.2-year follow-up
No. cases/N	666/2487	278/1121	157/699
**LE8 Component**			
Diet	0.96 (0.93; 0.99)**	0.98 (0.93; 1.02)	0.97 (0.92; 1.04)
Physical activity	0.93 (0.91; 0.95)***	0.94 (0.91; 0.97)***	0.95 (0.91; 1.00)*
Nicotine exposure	1.02 (0.98; 1.05)	0.97 (0.92; 1.02)	0.92 (0.87; 0.98)**
Sleep	0.95 (0.92; 0.99)**	0.96 (0.91; 1.01)	0.96 (0.89; 1.03)
BMI	0.89 (0.86; 0.92)***	0.96 (0.90; 1.01)	0.89 (0.83; 0.96)**
Blood lipids	1.01 (0.97; 1.05)	1.01 (0.95; 1.07)	1.04 (0.96; 1.13)
Blood glucose	0.93 (0.90; 0.96)***	0.93 (0.88; 0.98)**	0.94 (0.87; 1.01)
Blood pressure	0.99 (0.96; 1.02)	0.96 (0.92; 1.01)	0.97 (0.91; 1.03)

Note. LE8: Life's Essential 8; SPPB: Short Physical Performance Battery.

Logistic regression model adjusted for age, sex, education, time watching TV, energy intake, alcohol consumption, cardiovascular disease, respiratory disease, musculoskeletal disease, cancer and depression.

*p<0.05; **p<0.01; ***p<0.001

In line with our findings, a cross-sectional study on United States adults found inverse relationships between CVH, as estimated by the LE8 score, and sarcopenia, an important geriatric syndrome related to ILEF [27].Similarly, a cross-sectional analysis of Chilean older adults found that a higher LS7 score was associated with lower risk of disability, as measured by the World Health Survey [28], where mobility limitations, including those related to the lower extremity, were part of the score. Moreover, in a prospective study on Italian community dwelling older adults, a higher LS7 score was linked to decreased risk of disability in basic or instrumental activities of daily living over a 9 year follow-up [29]. These findings go in line with our own, as many of these disabilities may result from ILEF, and the association persisted during a long follow-up period. Further, a prospective study on Spanish community dwelling older adults free of CVD observed a relationship between ideal CVH and a reduced risk of frailty over 3.5 years [9]. Lastly, in a prospective study on postmenopausal women from the United States, a linear, dose-response relationship was found between higher SPPB scores and decreased CVD and CVD mortality risk, adding evidence to the intercorrelation between lower-extremity function and CVH [30].

Our results showed an inverse linear association between LE8 scores and the risk of low performance in the balance test, gait speed test, and chair-stand test. We also found this association for the incidence of low balance and low gait speed at both the 2.4-year and 5.2-year follow-ups. These findings are relevant for several reasons. First, gait speed is a well-established indicator of mobility, functional ability, and overall health, and serves as an important clinical marker for geriatric conditions such as sarcopenia and frailty. Low gait speed has also been linked to an increased risk of falls, cognitive decline, hospitalization, disability, and mortality [31]. Second, a good balance is essential for maintaining functional independence and preventing falls [32]. Balance reflects the integration of multiple physiological systems, including vision, proprioception, the vestibular system, central nervous system processing, and muscle strength [33]. Third, muscle power, assessed in this study through the chair-stand test, is a strong predictor of functional disability and mortality [34]. Consequently, promoting CVH may help prevent the development of several important geriatric syndromes.

The inverse association between the overall LE8 score and ILEF is consistent with the results observed for several of the LE8 components; indeed, physical activity and blood glucose predicted ILEF at the 2.4-year follow-up, while physical activity, nicotine exposure, and BMI did so at the 5.2-year follow-up. It is worth noting that physical activity's inverse relationship with ILEF was maintained across all three time points, consistent with the evidence that physical activity is a very strong protective factor for declining musculoskeletal health, functional limitation and disability [35]. Moreover, not engaging in physical activity [36], smoking [37], being overweight or obese [38], and having diabetes [39] have all been associated with increased risk of disability and/or sarcopenia, conditions related to ILEF.

Regarding blood lipids and blood pressure, no association was found between better scores in these components and a lower risk of ILEF in either the cross-sectional or longitudinal analyses. It can be speculated that this may be due to the widespread and typically effective treatment of these conditions, which often leads to normalization of measured values regardless of underlying disease. As a result, individuals with well-controlled hypertension or dyslipidemia may appear similar to those without the condition. Furthermore, LE8 adjustment by subtracting only 20 points in treated individuals may not sufficiently account for these differences, potentially attenuating the observed associations.

Strengths of this study are its prospective nature, which provides evidence supporting the hypothesis of a causal relationship between poor CVH and ILEF, and the use of validated measures of most variables. A limitation is that diet, physical activity, and chronic diseases were self-reported, potentially leading to measurement errors. However, the dietary history used in this study has been proved to have good validity and reproducibility [16]. Moreover, self-reported diet data of this type have been widely used in epidemiological studies, showing good reliability and validity as a predictor of many health outcomes [14]. Additionally, in this same cohort, self-reported physical activity has previously shown a similar association with unhealthy aging as physical activity measured by accelerometry [19]. Another limitation is that being diagnosed with a chronic condition may have led to changes in health behaviors and factors. In our study, CVD, musculoskeletal disease, and depression were observed in a higher proportion of individuals with ILEF. This is consistent with the fact that ILEF, as part of the functional disability geriatric syndrome, is interconnected and shares risk factors with other geriatric syndromes such as depression, which in turn leads to multimorbidity, with CVD being a frequent component [40]. Musculoskeletal disease leads directly to lower muscle strength and mass, which explains its higher prevalence within individuals with ILEF. However, as shown in Supplementary Figures 3, 4 and 5, the protective associations of CVH with ILEF were present, and showed some tendency to be stronger, in individuals without these chronic conditions, including CVD. Nevertheless, chronic disease onset did not appear to modify the association between baseline CVH and incident ILEF, which also remained similar when further adjusting for changes in the LE8 score over time. The potential attrition bias due to missing data or losses to follow-up may have attenuated the studied associations, as included participants were younger, had better habits and were generally healthier than those excluded and lost to follow-up. As a further limitation, despite adjusting for potential confounders, some residual confounding cannot be ruled out. Finally, this study was conducted on a Mediterranean population of older adults, who often exhibit healthier dietary patterns (Mediterranean diet), higher levels of physical activity, stronger social support networks, and better access to preventive care and chronic disease management compared to those in other regions of the world. This may limit the generalization of our findings to populations with different sociocultural or healthcare contexts.

In conclusion, a higher LE8 score was associated with both a lower prevalence and a lower incidence of ILEF in older adults. Accordingly, a comprehensive evaluation of CVH offers insight into older adults' current lower-extremity function and how it may progress over time, identifying opportunities for early intervention.

Acknowledgements

## Supplementary Materials

The Supplementary data can be found online at: www.aginganddisease.org/EN/10.14336/AD.2025.0347.


