# Supplementary material for: Cardiovascular Health, as per Life's Essential 8, and Impaired Lower-Extremity Function in Older Adults
Source: Aging Dis. 2025 Jun 13;17(4):2231–40. doi: 10.14336/AD.2025.0347 (PMC13256512; doi:10.14336/AD.2025.0347)
Supplement: Supplementary file 1 [file AD-17-4-2231-s.pdf]

Cardiovascular Health, as per Life's Essential 8, and Impaired Lower-Extremity Function in Older Adults

David Gómez-Ángel, Mercedes Sotos-Prieto, David Martínez-Gómez, Auxiliadora Graciani, Esther García-Esquinas, Fernando Rodríguez-Artalejo, Rosario Ortolá

Supplementary Table 1. Calculation of the Life's Essential 8 in the Seniors-ENRICA-2 cohort

**Domain**	**CVH metric**	**Method of measurement**	**Life's Essential 8 score**	
Health behaviors	Diet	Self-reported daily intake: The Alternative Healthy Eating Index-2010 (AHEI-2010)	Points	Scoring
			0	0-24th percentile
			25	25th – 49th percentile
			50	50th – 74th percentile
			80	75th – 95th percentile
			100	≥95th percentile
	Physical activity	Self-reported minutes of moderate and vigorous PA per week: EPIC Physical Activity Questionnaire	0	<1
			20	1–29
			40	30–59
			60	60–89
			80	90–119
			90	120–149
			100	≥150
	Nicotine exposure[a]	Self-reported use of cigarettes: telephone interview	0	Current smoker
			25	Former smoker, quit <1 y
			50	Former smoker, quit 1–<5 y
			75	Former smoker, quit ≥5 y
			100	Never smoker
	Sleep health[b]	Self-reported average hours of sleep per night: telephone interview	0	<4
			20	4–<5
			40	5–<6 or ≥ 10
			70	6–<7
			90	9–<10
			100	7–<9
Health factors	Body mass index	Body weight divided by height squared: Physical exam at home visit	0	≥ 40.0
			15	35.0-39.9
			30	30.0-34.9
			70	25.0-29.9
			100	<25
	Blood lipids (non-HDL cholesterol)[b]	Plasma total and HDL cholesterol with calculation of non-HDL cholesterol at first home visit	0	≥220
			20	190-219
			40	160-189
			60	130-159
			100	< 130
	Blood glucose[b]	FBG and HbA1 at first home visit	0	Diabetes with HbA1c≥10.0
			10	Diabetes with Hb A1c 9.0–9.9
			20	Diabetes with HbA1c 8.0–8.9
			30	Diabetes with HbA1c 7.0–7.9
			40	Diabetes with HbA1c <7.0
			60	No diabetes and FBG 100–125 (or HbA1c 5.7–6.4) (prediabetes)
			100	No history of diabetes and FBG <100 (or HbA1c <5.7)
	Blood pressure[b]	Mean of the 2 last measurements of casual BP	0	≥ 160 or ≥ 100
			25	140-159 or 90-99
			50	130-139 or 80-89
			75	120-129/<80
			100	<120/<80

Note. CVH, cardiovascular health; AHEI, Alternate Healthy Eating Index 2010; PA, physical activity; HDL, high density lipoprotein; FBG, fasting blood glucose.

[a] 20 points subtracted for living with an active indoor smoker at home.

[b] 20 points subtracted if drug treated.

SUPPLEMENTARY DATA

Supplementary Table 2. Comparison of baseline characteristics between participants included and those excluded or lost to follow-up

	**Cross-sectional analysis**		**5.2-year prospective analysis**	
	**Included**	**Excluded**	**Included**	**Lost to follow-up**
**N**	2,487	786	713	2,560
**Age (years)**	71.54 (4.38)	72.84 (4.68)*	71.13 (3.95)	72.05 (4.61)*
**Sex, n (%)**				
Male	1,173 (47.17)	363 (46.18)	355 (49.79)	1,181 (46.13)
Female	1,314 (52.83)	423 (53.82)	358 (50.21)	1,379 (53.87)
**Educational level, n (%)**				
Primary or less	1,581 (63.57)	532 (67.86)	390 (54.70)	1,723 (67.36)*
Secondary	464 (18.66)	138 (17.60)	131 (18.37)	471 (18.41)
University	442 (17.77)	114 (14.54)	192 (26.93)	364 (14.23)
**Time watching TV (h/day)**	3.17 (1.56)	3.44 (1.78)*	2.99 (1.40)	3.31 (1.70)*
**Energy intake (kcal/day)**	1949 (354)	1975 (356)	1954 (322)	1952 (365)
**Alcohol consumption, n (%)**				
Never drinker	468 (18.82)	58 (19.14)	116 (16.27)	410 (19.74)*
Moderate drinker	1,318 (53.00)	165 (54.46)	378 (53.02)	1,105 (53.20)
Heavy drinker	544 (21.87)	53 (17.49)	187 (26.23)	410 (19.74)
Former drinker	157 (6.31)	27 (8.91)	32 (4.49)	152 (7.32)
**Cardiovascular disease diagnosis, n (%)**	83 (3.34)	76 (9.67)*	19 (2.66)*	140 (5.47)*
**Respiratory disease diagnosis, n (%)**	194 (7.80)	58 (7.38)	54 (7.57)	198 (7.73)
**Musculoskeletal disease diagnosis, n (%)**	1,120 (45.03)	350 (44.53)	295 (41.37)	1,175 (45.90)*
**Cancer diagnosis, n (%)**	71 (2.85)	29 (3.69)	22 (3.09)	78 (3.05)
**Depression diagnosis, n (%)**	204 (8.20)	75 (9.54)	49 (6.87)	230 (8.98)
**ILEF (SPPB ≤ 9), n (%) [n]**	666 (26.78)	140 (41.54)*	146 (20.51)	660 (31.25)*

Note. ILEF, impaired lower extremity function; SPPB, Short Physical Performance Battery. Values are means (standard deviations) unless otherwise specified.
For excluded participants from the cross-sectional analysis, time watching TV was missing in 5 individuals, energy intake and alcohol consumption in 483, and the SPPB in 449.
*p<0.05 for the comparison with participants included in the analysis using Chi squared tests for categorical variables and Student's t-tests for continuous variables.

SUPPLEMENTARY DATA

Supplementary Table 3. Odds ratios (95% CI) for the association between baseline LE8 and incident impaired lower-extremity function (SPPB ≤9), stratified by incident chronic disease status at 2.4 and 5.2 years of follow-up.

	No. cases/N	Odds ratio (95% CI)	p-value
**2.4-year prospective**			
Incident chronic disease	85/273	0.75 (0.60; 0.94)	0.013
Non-incident chronic disease	193/848	0.79 (0.69; 0.91)	0.001
Incident chronic disease x LE8			0.948
**5.2-year prospective**			
Incident chronic disease	54/192	0.65 (0.50; 0.84)	0.001
Non-incident chronic disease	103/507	0.84 (0.69; 1.02)	0.077
Incident chronic disease x LE8			0.179

Note. LE8: Life's Essential 8; SPPB: Short Physical Performance Battery.
Incident chronic disease was defined as developing either cardiovascular disease, respiratory disease, musculoskeletal disease, cancer or depression during the follow-up period.
Logistic regression model adjusted for baseline age, sex, education, time watching TV, energy intake, alcohol consumption, and LE8 × incident chronic disease interaction.

Supplementary Table 4. Odds ratios (95% confidence interval) for the 2.4-year prospective and 5.2-year prospective association of LE8 score with impaired lower-extremity function (SPPB ≤9), with additional adjustment for change in LE8 score.

	Low LE8	Moderate LE8	High LE8	p-trend	Per 10 points
**2.4 year prospective**					
No. cases/N	36/118	210/832	17/128		263/1078
Model 4	Ref.	0.65 (0.40; 1.06)	0.22 (0.10; 0.45)***	<0.001	0.70 (0.61; 0.80)***
**5.2 year prospective**					
No. cases/N	21/57	100/425	13/84		134/566
Model 4	Ref.	0.46 (0.23; 0.90)*	0.20 (0.08; 0.53)**	0.001	0.67 (0.55; 0.82)***

Note. LE8: Life's Essential 8. SPPB, Short Physical Performance Battery.
Model 4: Logistic regression model adjusted for: age, sex, education, time watching TV, energy intake, alcohol consumption, cardiovascular disease, respiratory disease, musculoskeletal disease, cancer, depression and change in LE8 score between baseline and each follow-up.
Follow-up data on LE8 were missing for 43 participants at the 2.4-year time point and for 133 participants at the 5-2-year follow-up.
*p<0.05; **p<0.01; ***p<0.001

© 2025. Gómez-Ángel D et al. Published online at http://www.aginganddisease.org/EN/10.14336/AD.2025.0347

SUPPLEMENTARY DATA

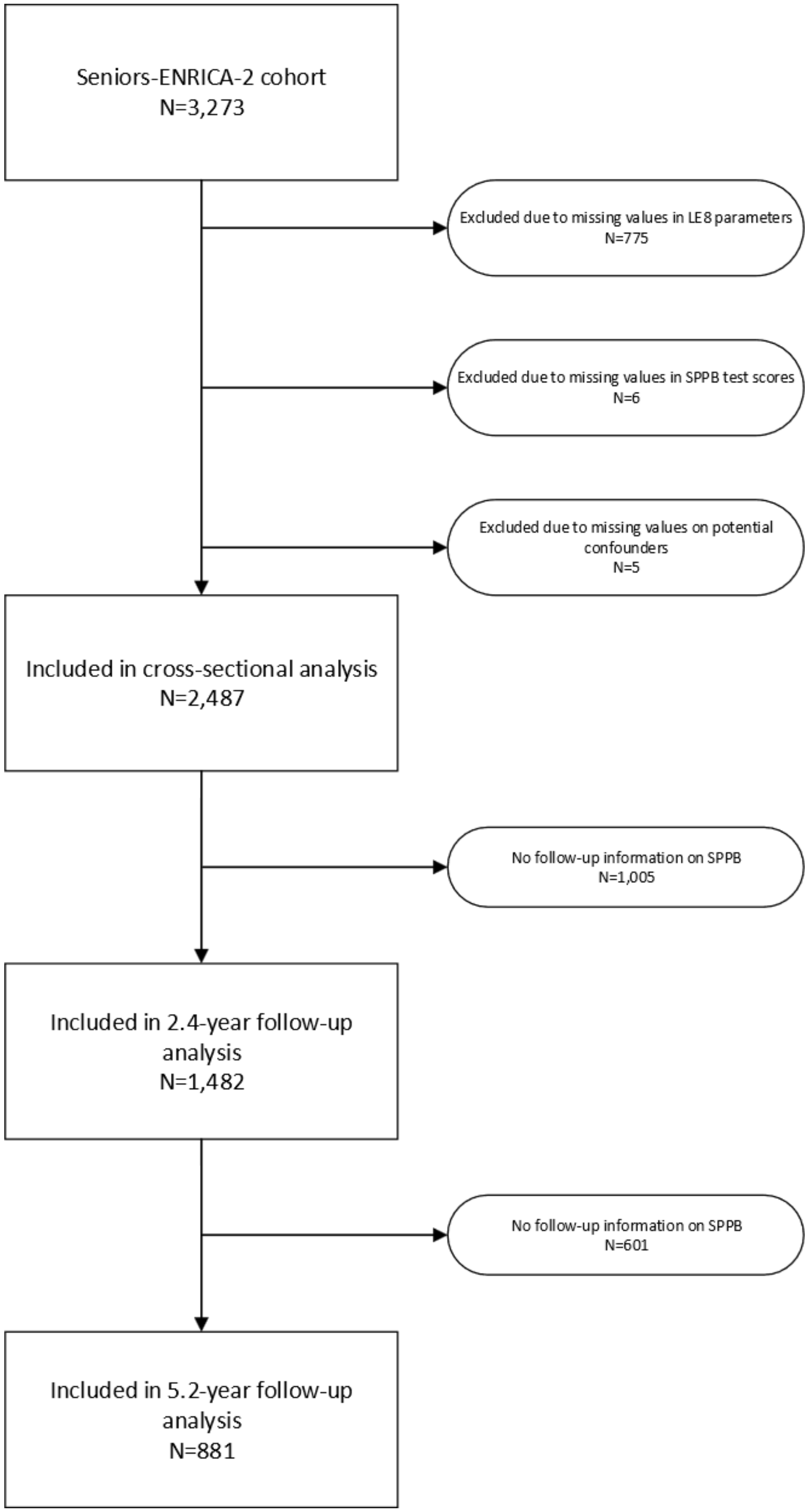

Supplementary Figure 1. Flowchart showing the number of participants included in each analysis

© 2025. Gómez-Ángel D et al. Published online at http://www.aginganddisease.org/EN/10.14336/AD.2025.0347

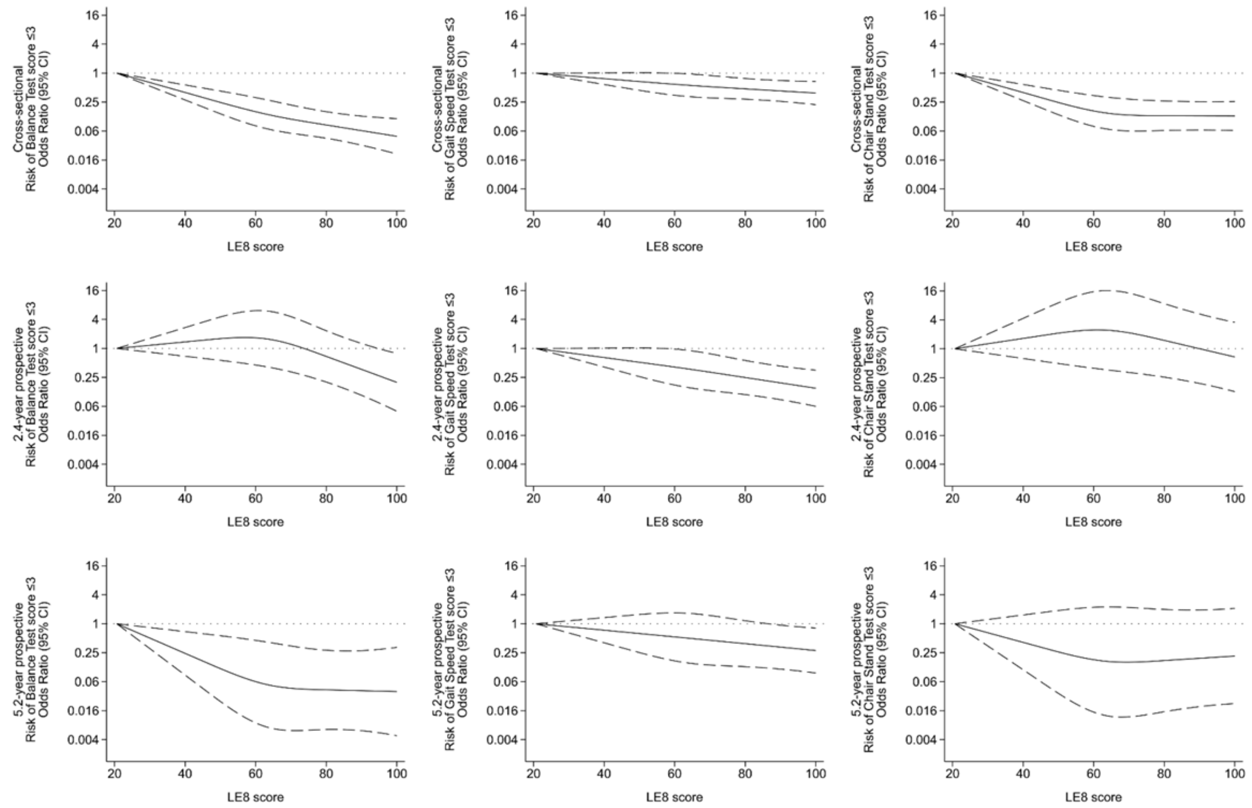

Supplementary Figure 2. Dose-response association of global Life's Essential 8 score (LE8) with low score in the individual SPPB components. Restricted cubic spline logistic regression model adjusted for age, sex, education, time watching TV, energy intake, alcohol consumption, cardiovascular disease, respiratory disease, musculoskeletal disease, cancer and depression.

© 2025. Gómez-Ángel D et al. Published online at http://www.aginganddisease.org/EN/10.14336/AD.2025.0347

SUPPLEMENTARY DATA

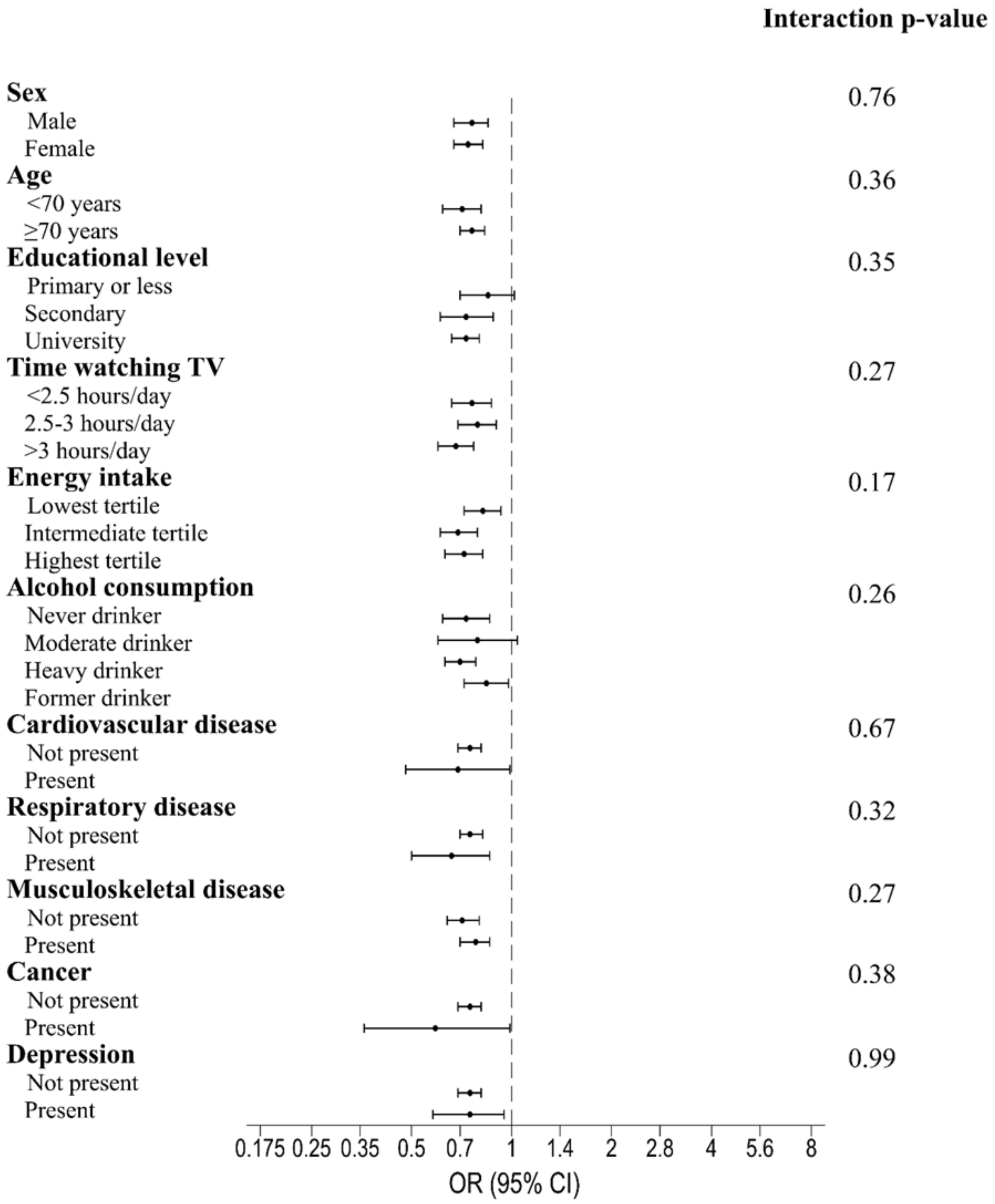

Supplementary Figure 3. Odds ratios (95% confidence intervals) for the cross-sectional association between Life's Essential 8 and impaired lower-extremity function by categories of sociodemographic, lifestyle, and clinical variables.

© 2025. Gómez-Ángel D et al. Published online at http://www.aginganddisease.org/EN/10.14336/AD.2025.0347

SUPPLEMENTARY DATA

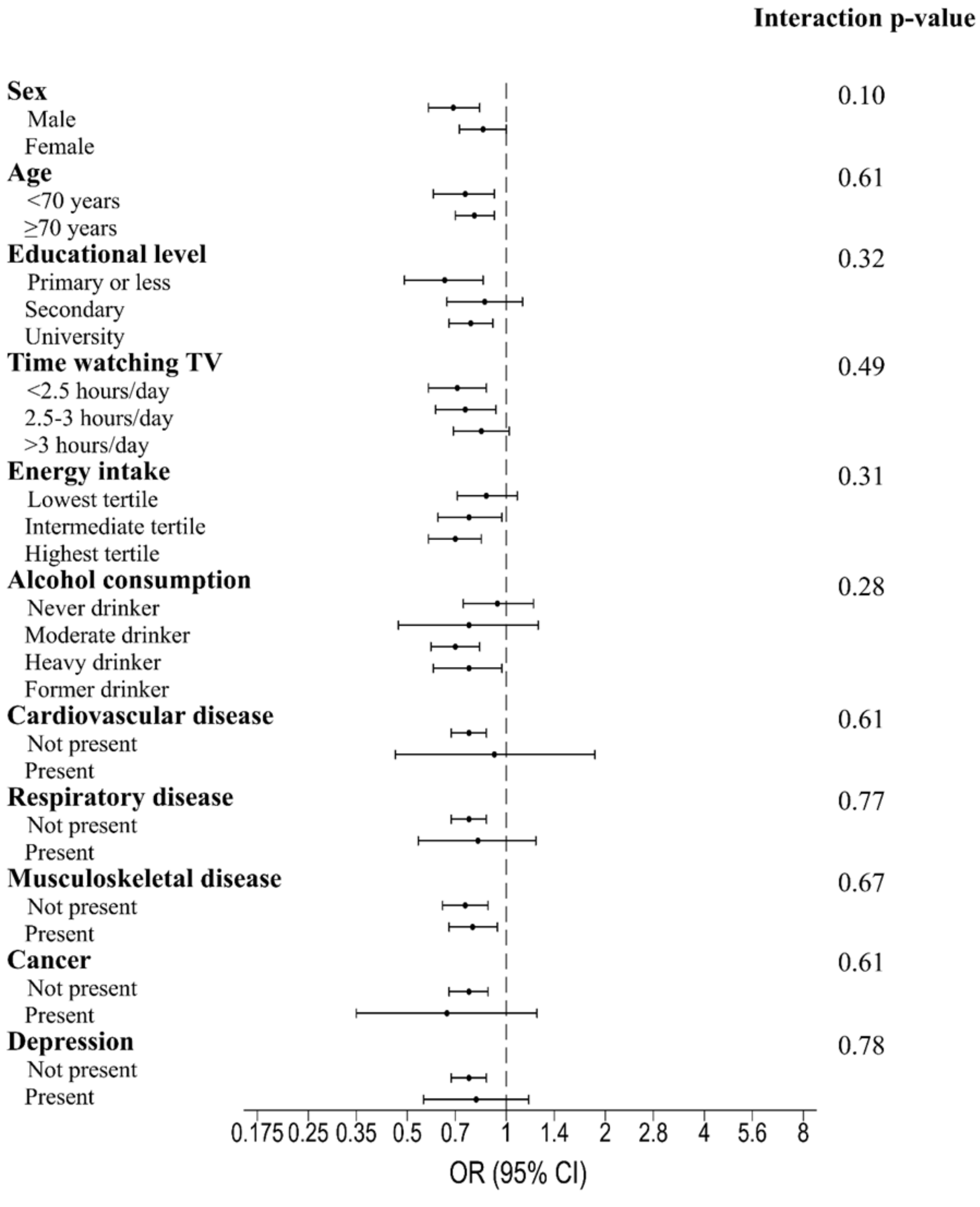

Supplementary Figure 4. Odds ratios (95% confidence intervals) for the 2.4-year prospective association between Life's Essential 8 and impaired lower-extremity function by categories of sociodemographic, lifestyle,and clinical variables.

© 2025. Gómez-Ángel D et al. Published online at http://www.aginganddisease.org/EN/10.14336/AD.2025.0347

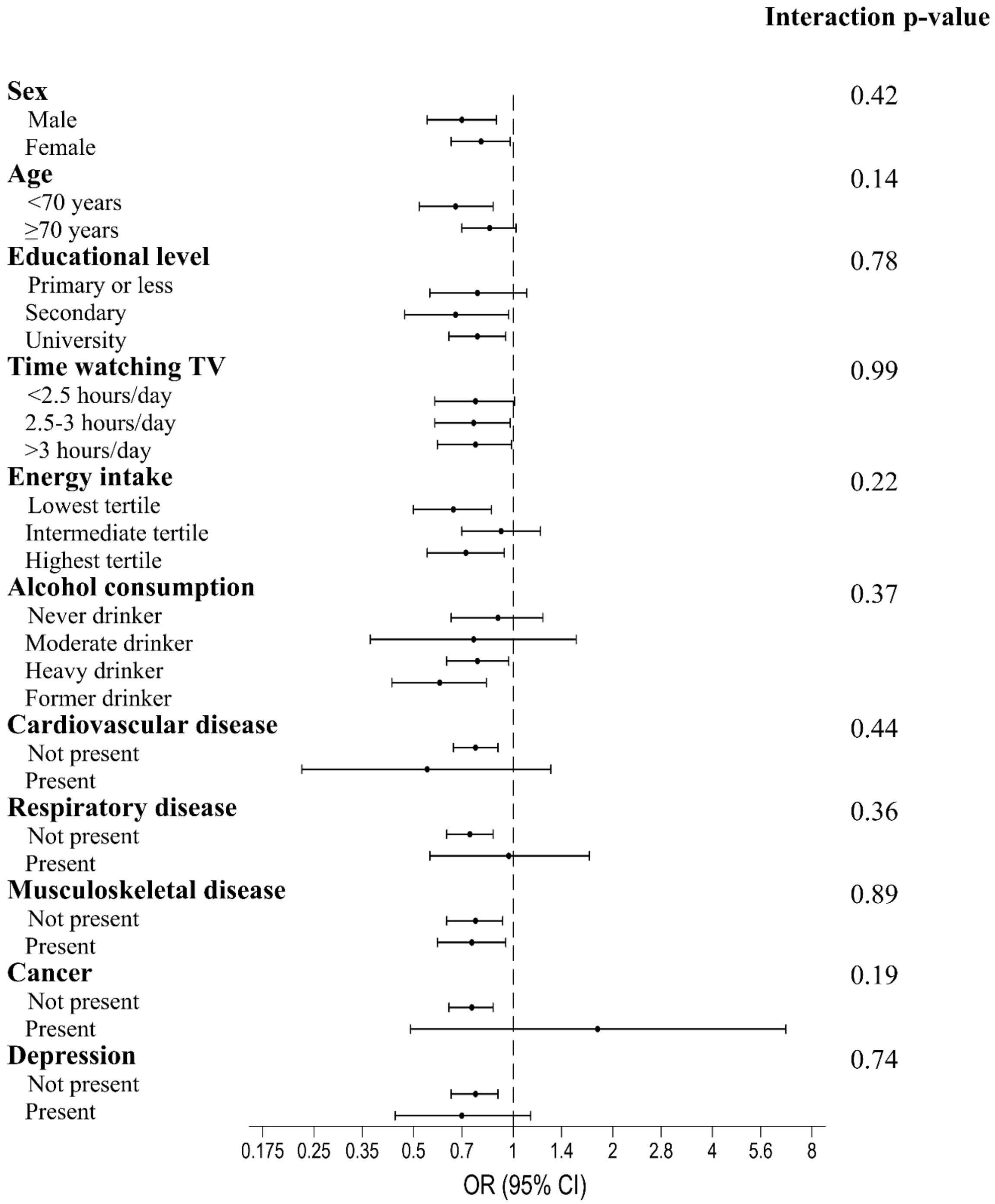

Supplementary Figure 5. Odds ratios (95% confidence intervals) for the 5.2-year prospective association between Life's Essential 8 and impaired lower-extremity function by categories of sociodemographic, lifestyle, and clinical variables.

© 2025. Gómez-Ángel D et al. Published online at http://www.aginganddisease.org/EN/10.14336/AD.2025.0347
